# Ferulic Acid in the Treatment of Papulopustular Rosacea: A Randomized Controlled Study

**DOI:** 10.1111/jocd.16611

**Published:** 2024-10-16

**Authors:** Xing Wang, Yonghong Xue, Hongzi Zhu, Jingjie Zhang, Meiling Li, Wenxiu Ge, Zengxiang Luo, Xiangfeng Yuan, Dong Zhang, Weiyuan Ma

**Affiliations:** ^1^ Department of Dermatology Affiliated Hospital of Shandong Second Medical University Weifang Shandong China; ^2^ Yantai Xianse Trading Co., Ltd. Yantai Shandong China

**Keywords:** efficacy, ferulic acid, papulopustular type, rosacea, skin barrier

## Abstract

**Background:**

Rosacea is a chronic inflammatory skin disease characterized by flushing, erythema, papules, and pustules on the central face. It affects patient appearance and is noted for its chronicity, recurrence, and resistance to treatment. Effective rosacea treatment requires repairing the skin barrier, reducing inflammation, and promoting vasoconstriction.

**Aims:**

This study aims to evaluate the efficacy of topical ferulic acid in treating papulopustular rosacea and its impact on skin barrier function.

**Methods:**

Sixty patients with mild to moderate papulopustular rosacea were selected from the Department of Dermatology at the Affiliated Hospital of Shandong Second Medical University between January 2023 and December 2023. Patients were randomly assigned to either a control group or an observation group, with 30 patients in each group. The observation group applied ferulic acid solution to the affected areas, while the control group used normal saline, both twice daily for 6 weeks. Both groups also received 0.1 g doxycycline hydrochloride tablets orally once daily. Skin lesions and skin barrier function were assessed using VISIA imaging and self‐rating scales before and during treatment, and adverse reactions were recorded.

**Results:**

After 6 weeks, both skin lesion assessments and self‐assessment scores improved significantly from baseline, with greater improvement in the observation group compared to the control group (*p* < 0.05). Indicators of skin barrier function and VISIA imaging results demonstrated the efficacy of ferulic acid in treating rosacea. The total effective rate was significantly higher in the observation group (80.00%) compared to the control group (63.33%) (*p* < 0.05). In the observation group, nine patients (30.00%) experienced a greasy sensation initially, one patient (3.33%) reported tingling and itching, and no serious adverse reactions were observed.

**Conclusions:**

Ferulic acid is effective as an adjuvant treatment for papulopustular rosacea, significantly improving skin lesions and repairing skin barrier function with minimal adverse reactions.

## 1 Introduction

Rosacea is a chronic, recurrent inflammatory skin disease primarily affecting the center of the face, targeting facial blood vessels, nerves, hair follicles, and sebaceous glands [1]. According to a recent meta‐analysis of international epidemiological data, the global average incidence of rosacea is 5.5% [2]. The symptoms of rosacea are diverse and can severely impact patient appearance, including paroxysmal flushing, persistent erythema, telangiectasia, papules, and pustules on the facial skin. Additionally, a minority of patients may experience hyperplasia and ocular involvement [3]. Given the facial location of these skin lesions, the chronic nature, recurrence, and often unsatisfactory treatments of rosacea can significantly affect patients' mental health. Studies indicate that individuals with rosacea are prone to low self‐esteem, anxiety, depression, and social phobia, which can seriously impact their work, education, and personal lives [4]. Negative psychological emotions have been confirmed to exacerbate rosacea by interfering with the neuroendocrine system, creating a vicious cycle [5]. Overall, rosacea profoundly impacts patients' physical and mental health.

The pathogenesis of rosacea is not yet fully elucidated. It is generally believed that rosacea is a chronic inflammatory disease induced by multiple factors on a genetic background, characterized by abnormalities in natural immunity and neurovascular regulation. Disruptions in skin microecology, particularly the colonization of *Demodex folliculorum*, induce abnormal activation of the innate immune response, causing inflammatory cell infiltration around hair follicles and blood vessels. This leads to capillary dilation, sebaceous gland hyperplasia, and further damage to skin barrier function [6]. Skin barrier damage is a significant pathogenic factor of rosacea and contributes to skin sensitivity symptoms [7]. Therefore, based on the pathogenesis of rosacea, key treatment strategies include repairing skin barrier function, reducing inflammation, and promoting vasoconstriction.

Ferulic acid, a derivative of cinnamic acid, is naturally found in plants such as Ferula, *Angelica sinensis*, and *Ligusticum chuanxiong* [8]. It has been shown to have anti‐inflammatory, antioxidant, anti‐aging, depigmenting, and UV protective effects [9]. As an organic weak acid, ferulic acid also has the potential to repair damaged skin barrier function. Currently, there are no reports on the use of ferulic acid for treating rosacea. This study investigates the efficacy and adverse reactions of ferulic acid in treating papulopustular rosacea using clinical samples, aiming to establish a foundation for its application in managing this condition.

## 2 Materials and Methods

### 2.1 Study Participants

This randomized, single‐center, single‐blind, prospective controlled trial was approved by the Ethics Committee of the Affiliated Hospital of Shandong Second Medical University (approval number wyfy‐2023‐ky‐172) and conducted in accordance with the Declaration of Helsinki. Sixty patients with papulopustular rosacea, who visited the dermatology outpatient department at the Affiliated Hospital of Shandong Second Medical University between January and December 2023, were recruited and provided written informed consent.

The diagnosis of rosacea was based on the European Consensus (S2k) Guidelines for Rosacea [10]. Inclusion criteria were: papulopustular rosacea primarily on both cheeks, mild to moderate rash (Clinician Erythema Assessment [CEA] score 0–3 and Investigator Global Assessment of Rosacea Severity Score [IGA‐RSS] 0–4), age over 18 years, and no specific treatment within the past month. Exclusion criteria were: pregnant or lactating patients; those who had received oral or topical medication within the past month; patients with other facial skin diseases; those with infectious diseases, malignant tumors, cardiovascular disorders, mental illness, or communication disorders; and those allergic to tetracycline antibiotics.

### 2.2 Research Design

The 60 patients were randomly assigned to an observation group and a control group using a random number table. Both groups received oral doxycycline hydrochloride tablets (Kaifeng Pharmaceutical Group Co., Ltd., national medicine standard number H41020946, 0.1 g/tablet) at a dosage of 100 mg once daily. After cleansing their faces each morning and evening, the observation group applied a ferulic acid solution (Dissolve ferulic acid in polyols, then prepare it into a 3% solution using serine diglycol ester and adjust the pH to 5.0 [10 mL, Yantai Xianse Trading Co., Ltd]) to the affected areas, while the control group applied normal saline (China Otsuka Pharmaceutical Co., Ltd., National Medicine Standard No. H20043271, 10 mL: 0.09 g). The treatment duration was 6 weeks. Patients were instructed to keep their faces clean and protect themselves from sun exposure.

### 2.3 Efficacy Evaluation

#### 2.3.1 Clinical Skin Lesion Score

Clinical photographs (Nikon D7100) were taken at standardized positions before treatment and at 2, 4, and 6 weeks. Two blinded investigators scored these using the CEA (Table 1) and IGA‐RSS (Table 2).

**TABLE 1:** Clinician Erythema Assessment (CEA) and Patient Self‐Assessment (PSA).

Scores	CEA	PSA
0, Clear	Clear skin with no signs of erythema	Clear of unwanted redness
1, Almost clear	Almost clear with slight redness	Nearly clear of unwanted redness
2, Mild	Mild erythema with definite redness	Somewhat more redness than I prefer
3, Moderate	Moderate erythema with marked redness	More redness than I prefer
4, Severe	Severe erythema with fiery redness	Completely unacceptable redness

**TABLE 2:** IGA‐RS score.

Numerical score	Definition	Description
0	Clear	Almost no Rosacea (i.e., no papules and/or pustules); no residual erythema; mild to moderate degree of telangiectasia may be present
1	Minima	Rare papules and/or pustules; residual to mild erythema; mild‐to‐moderate degree of telangiectasia may be present; few papules and/or pustules; mild erythema; mild to moderate degree of telangiectasia may be present
2	Mild	Few papules and/or pustules; mild erythema; mild‐to‐moderate degree of telangiectasia may be present
3	Mild to moderate	Distinct number of papules and/or pustules; mild to moderate erythema; mild to moderate degree of telangiectasia may be present
4	Moderate	Pronounced number of papules and/or pustules; moderate erythema; mild to moderate degree of telangiectasia may be present
5	Moderate to severe	Many papules and/or pustules, occasionally with large inflamed lesions; moderate erythema; moderate degree of telangiectasia may be present
6	Severe	Numerous papules and/or pustules, occasionally with confluent areas of inflamed lesions; moderate to severe erythema; moderate to severe degree of telangiectasia may be present

#### 2.3.2 Skin Barrier Function

Skin surface hydration level (SSH) and transepidermal water loss (TEWL) were measured using a water loss tester (CK, Germany) before treatment and at 2, 4, and 6 weeks.

#### 2.3.3 VISIA Imaging Analysis

VISIA imaging of patients was conducted before treatment and at 2, 4, and 6 weeks.

#### 2.3.4 Patient Self‐Assessment

Patients self‐assessed their clinical photographs before treatment and at 2, 4, and 6 weeks (Table 1).

#### 2.3.5 Adverse Reactions

Adverse reactions during the treatment were recorded.

#### 2.3.6 Efficacy Evaluation

At the sixth week, efficacy was evaluated using the efficacy index, calculated as: (score before treatment—score after treatment)/score before treatment × 100%. Efficacy categories were: cured (efficacy index ≥ 90%), markedly effective (50% ≤ efficacy index < 90%), improved (30% ≤ efficacy index < 50%), and ineffective (efficacy index < 30%). The effective rate was defined as (number of cured and markedly effective cases/total number of cases) × 100%.

### 2.4 Statistical Methods

Data were analyzed using SPSS 26.0 statistical software. Subjective and objective scores and other normally distributed data are presented as mean ± standard deviation (mean ± SD). Independent sample *t*‐tests were used for intergroup comparisons, and paired *t*‐tests for intragroup comparisons. Enumeration data are presented as numbers and percentages, compared using the *χ* ^*2*^ test. The rank‐sum test was used for comparing two groups of independent graded data. Differences were considered statistically significant at *p* < 0.05.

## 3 Results

### 3.1 Comparison of General Data Between the Two Groups

This study included 60 patients with papulopustular rosacea, all of whom completed the treatment regimen. The cohort comprised 11 males and 49 females, aged 26–53 years (mean age: 39.50 ± 8.79 years), with a disease duration of 0.5–7 years (mean duration: 2.61 ± 1.94 years). The patients were randomly assigned to the observation group or the control group, with 30 patients in each. The observation group consisted of seven males and 23 females, aged 27–53 years, with a mean age of 39.14 ± 9.12 years and a disease duration of 0.5–6 years (mean: 2.79 ± 1.87 years). The control group consisted of five males and 25 females, aged 26–52 years, with a mean age of 39.86 ± 9.15 years and a disease duration of 1–7 years (mean: 2.43 ± 2.15 years). There were no significant differences in sex, age, or disease duration between the two groups (*p* > 0.05) (Table 3).

**TABLE 3:** Comparison of basic data between the observer and the control group.

	Observer	Control	*χ* ^2^/*t*	*p*
Example (Num.)	30	30		
Gender (male/female)	7/23	5/25	0.417	0.519
Age (years)	39.14 ± 9.12	39.86 ± 9.15	0.146	0.886
Disease duration (years)	87.32 ± 13.66	64.36 ± 10.03	0.332	0.746

### 3.2 Comparison of Clinical Skin Lesion Scores Before and After Treatment

**FIGURE 1:**
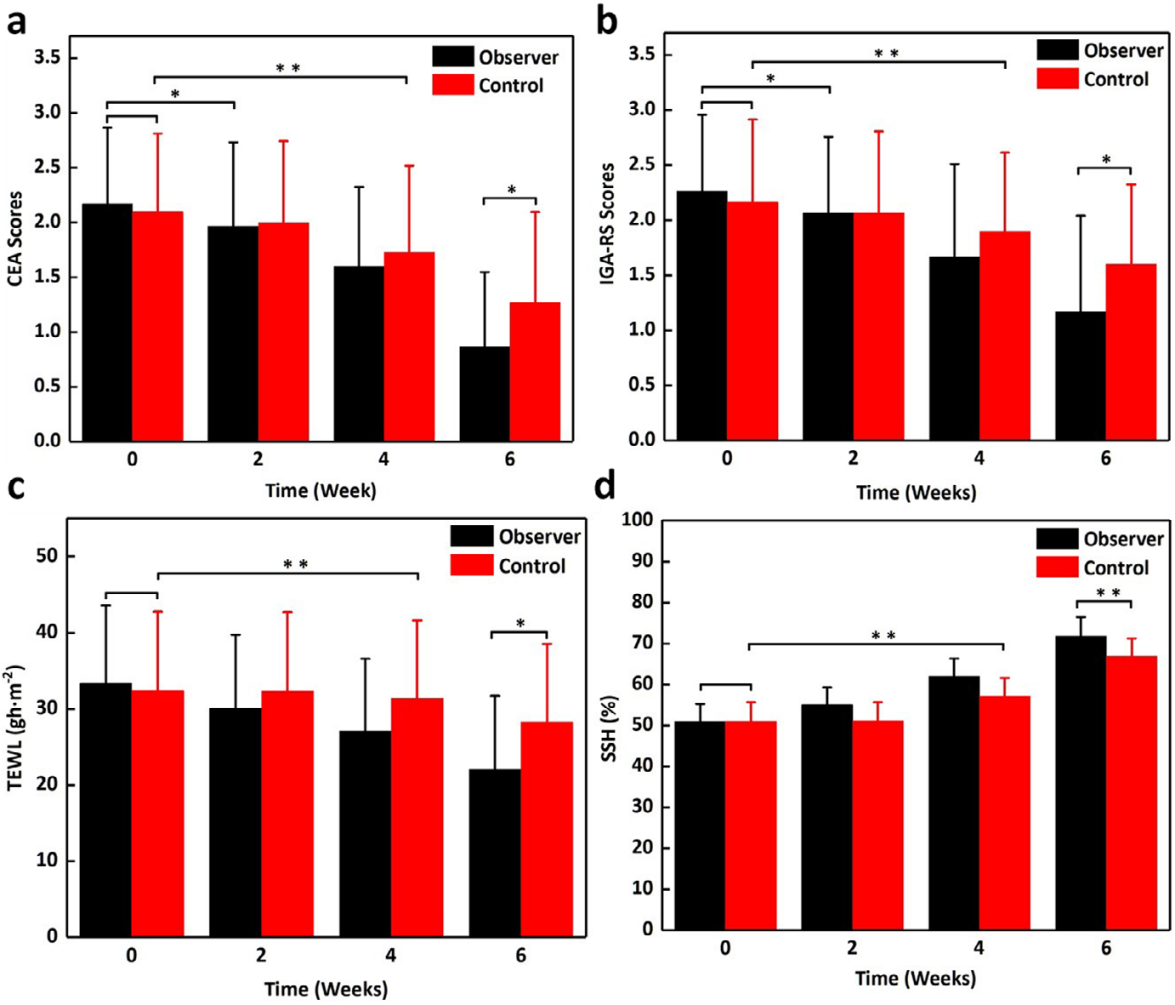
The comparison of CEA scores (a), IGA‐RS scores (b), SSH (c), and TEWL (d) between the observer and the control group during treatment. Data are presented as mean ± SD (standard error) of three parallel experiments. *P < 0.05, **P < 0.01.

Before treatment, there were no significant differences in the CEA or IGA‐RSS scores between the two groups (*t* = 0.366, *p* = 0.716; *t* = 0.538, *p* = 0.592, respectively). In the control group, significant improvements were observed from the fourth week onward (*t* = 4.097, *p* < 0.01; *t* = 3.247, *p* = 0.003, respectively). In the observation group, significant improvements were noted from the second week onward (*t* = 2.693, *p* = 0.012; *t* = 2.693, *p* = 0.012, respectively). At the sixth week, the scores of the observation group were significantly lower than those of the control group (*t* = 2.044, *p* = 0.046; *t* = 2.091, *p* = 0.041, respectively) (Figure 1a,b).

### 3.3 Comparison of Skin Barrier Function Before and After Treatment

Before treatment, there were no significant differences in SSH or TEWL between the two groups (*t* = 0.033, *p* = 0.974; *t* = 0.376, *p* = 0.709, respectively). In the control group, significant improvements were observed from the fourth week onward (*t* = 95.489, *p* < 0.01; *t* = 11.645, *p* < 0.01, respectively). In the observation group, significant improvements were noted from the second week onward (*t* = 49.962, *p* < 0.01; *t* = 18.438, *p* < 0.01, respectively). At the sixth week, TEWL in the observation group was significantly lower than in the control group (*t* = 2.441, *p* = 0.018), and SSH in the observation group was significantly higher than in the control group (*t* = 4.214, *p* < 0.01) (Figure 1c,d).

### 3.4 Comparison of VISIA Images Before and After Treatment in the Observation Group

**FIGURE 2:**
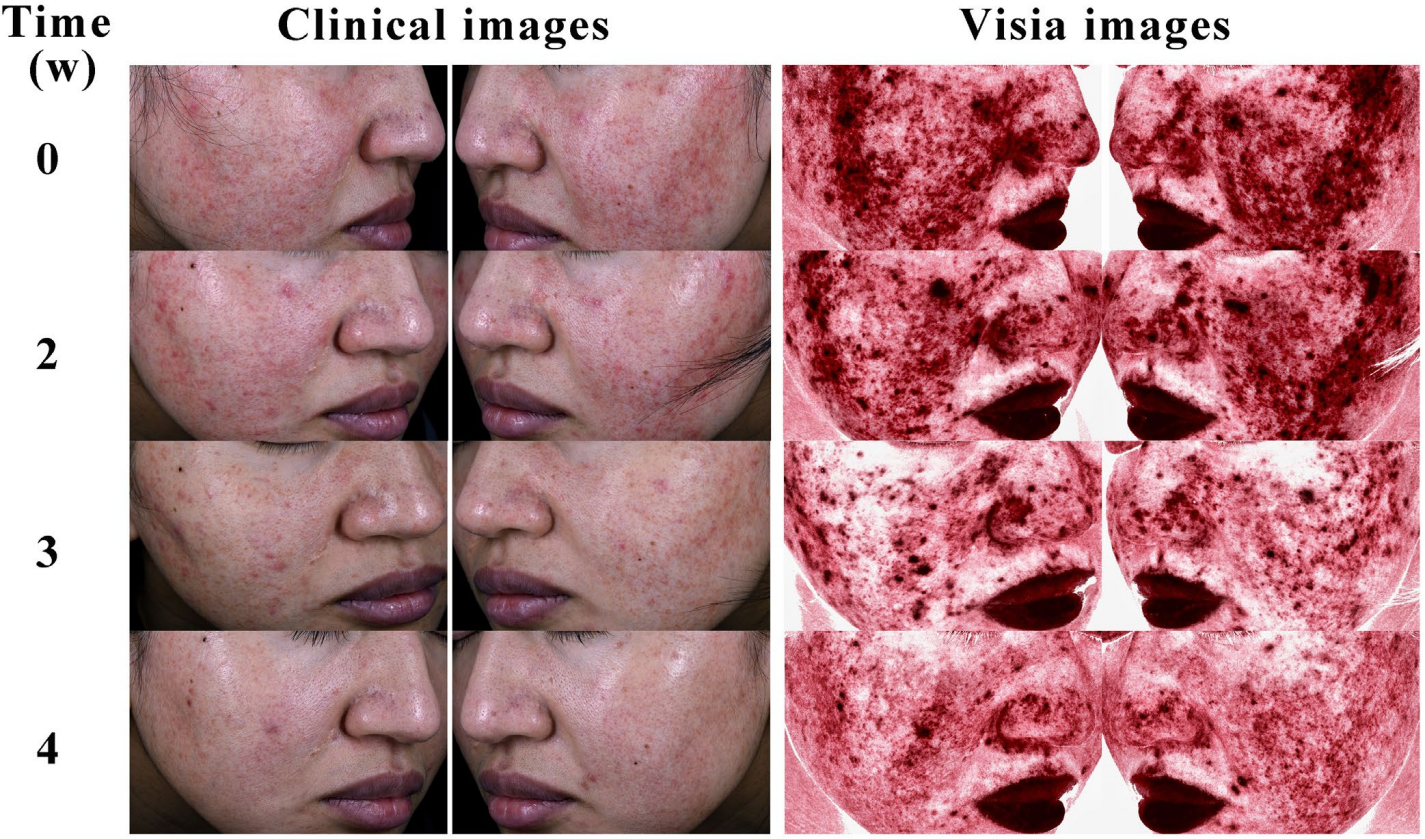
Comparison of typical case outcomes in the observation group before and after treatment: A 21‐year‐old female with a 1‐year history of moderate inflammatory rosacea.

**FIGURE 3:**
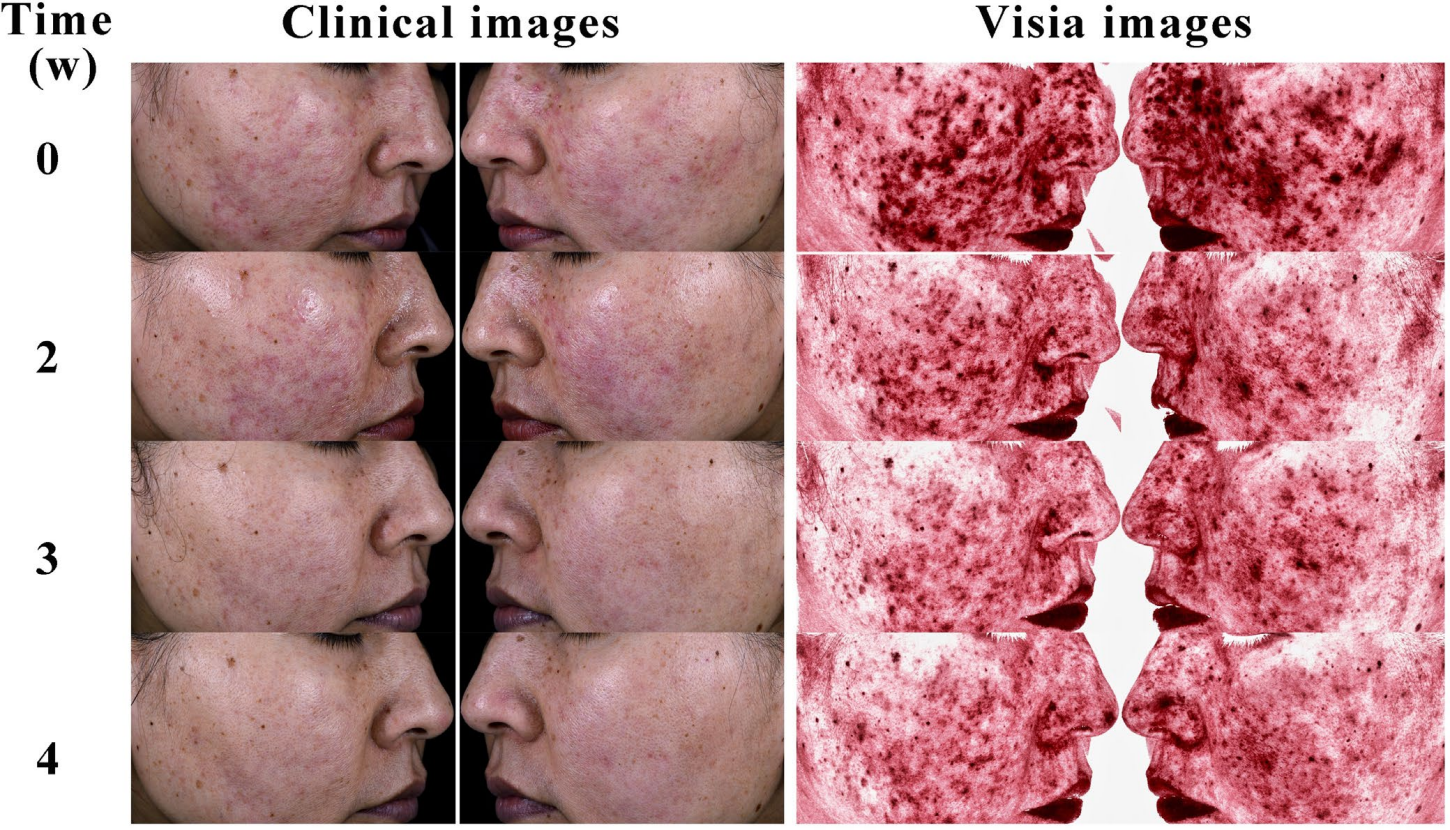
Comparison of treatment effects in a 38‐year‐old female with a 3‐year history of moderate inflammatory rosacea: pre‐ and post‐treatment analysis.

Images of skin lesions from two female patients, aged 38 and 21, were collected. The clinical images include standard photographs of the lesions, while VISIA images highlight inflammation features. Images were taken at the beginning of the treatment and at the second, fourth, and sixth weeks of treatment, labeled as 0, 2, 4, and 6, respectively. Initially, both patients exhibited erythema, papules, and pustules on the nose and cheeks. Post‐treatment, there was a notable improvement in the skin lesions, with reduced erythema and fewer papules and pustules (Figures 2 and 3).

### 3.5 Comparison of Patient Self‐Assessment Scores Before and After Treatment

**FIGURE 4:**
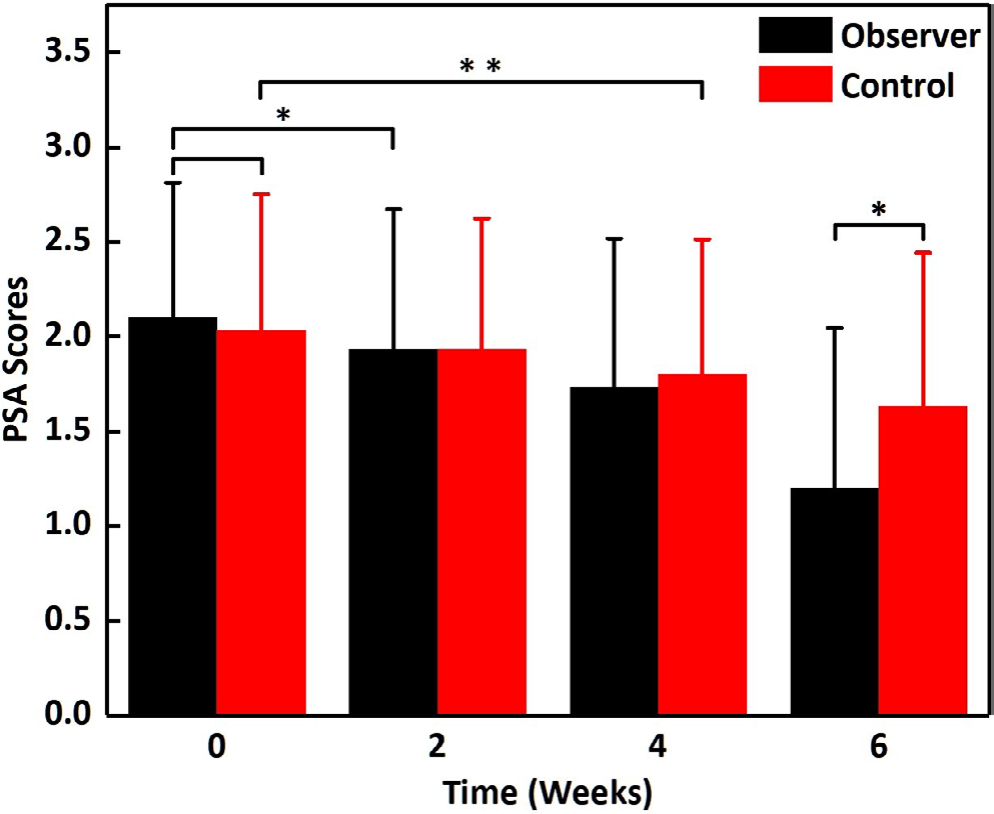
The comparison of PSA scores between the observer and the control group during treatment. Data are presented as mean ± SD (standard error) of three parallel experiments. *P < 0.05, **P < 0.01.

Before treatment, there were no significant differences in patient self‐assessment scores between the two groups (*t* = 0.361, *p* = 0.719). In the control group, significant improvements were observed from the fourth week onward (*t* = 2.971, *p* = 0.006). In the observation group, significant improvements were noted from the second week onward (*t* = 2.408, *p* = 0.023). At the sixth week, the self‐assessment scores of the observation group were significantly lower than those of the control group (*t* = 2.027, *p* = 0.047) (Figure 4).

### 3.6 Adverse Reactions

In the observation group, nine patients (30.00%) reported a greasy sensation during initial treatment, and one patient (3.33%) experienced tingling and itching, which subsided after tolerance was established. The remaining patients had no significant adverse reactions.

### 3.7 Comparison of Therapeutic Effects Between the Two Groups

The total effective rate in the observation group was 80.00%, compared to 63.33% in the control group. The difference in total effective rates between the groups was statistically significant (*χ* ^*2*^ = 2.060, *p* = 0.039), as shown in Table 4. Typical cases are illustrated in Figures 3 and 4.

**TABLE 4:** Comparison of before and after treatment between the observer and the control group.

	Observer	Control
Example (Num.)	30	30
Cure (Num./%)	9 (30.00)	4 (13.33)
Effective (Num./%)	15 (50.00)	15 (50.00)
Improve (Num./%)	4 (13.33)	6 (20.00)
Invalid (Num./%)	2 (6.67)	5 (16.67)
Total effective (Num./%)	24 (80.00)	19 (63.33)

## 4 Discussion

Rosacea is a multifactorial disease with a pathogenesis that remains not fully understood. Triggering factors include abnormalities in innate immunity, inflammatory responses to skin microorganisms, ultraviolet (UV) damage, and vascular dysfunction [10]. Hypotheses suggest that innate immune abnormalities and neurovascular dysregulation are primary drivers of rosacea pathogenesis [11]. Dysfunction in the innate immune system can lead to chronic inflammation and vascular issues, with antimicrobial peptides acting as triggers. LL37, the only cathelicidin family antimicrobial peptide identified in humans, plays a critical role in immune defense, cell chemotaxis, and angiogenesis [12]. In rosacea lesions, the expression and activity of kallikrein 5 in the stratum corneum are elevated, resulting in increased LL37 levels due to the cleavage of hCAP18 [13]. Injection of LL37 into mice induces erythema and blood vessel dilation, creating a rosacea‐like dermatitis model, highlighting LL37's pivotal role in rosacea inflammation [14]. LL37 upregulates TRPV4 expression in mast cells via the G protein‐coupled receptor MRGX2, leading to increased cation influx, sustained mast cell degranulation, and release of inflammatory mediators [15]. LL37 also activates the NLRP3 inflammasome in macrophages through the purinergic receptor P2X7, leading to the activation of caspase‐1 and interleukin (IL)‐1β, and recruitment of neutrophils and monocytes to the skin [16]. Additionally, Toll‐like receptor 2 in keratinocytes binds LL37, activating the mammalian target of rapamycin complex 1 (mTORC1) pathway, which upregulates LL37 expression and promotes the production of rosacea‐related chemokines and cytokines, thus exacerbating inflammation [17]. Dysregulated commensal bacteria in the skin of rosacea patients overexpress LL37, stimulating plasmacytoid dendritic cells (pDCs) to produce type I interferon (IFN), which promotes angiogenesis [18].

The treatment of rosacea aims to alleviate skin lesions, such as flushing, erythema, pustules, and papules, as well as symptoms like itching, burning, and pain. Treatment modalities include skincare, cosmetics, topical and oral medications, phototherapy, and injections [19]. Clinical treatments include ivermectin and tetracycline antibiotics to reduce *Propionibacterium* acnes and *Demodex* mite colonization. Brimonidine, oxymetazoline, carvedilol, and pulsed dye lasers are used to improve flushing and erythema, while carbon dioxide or erbium lasers remove hyperplastic tissue in nasal rosacea. However, these treatments pose challenges: minocycline can cause pigmentation and dizziness, brimonidine can induce rebound erythema, carvedilol can cause bradycardia and hypotension, and laser treatments can further damage the skin barrier. Therefore, the development of new treatments for rosacea is essential.

Research indicates that ferulic acid offers significant therapeutic effects on the skin. It exhibits anti‐inflammatory properties by inhibiting the expression of cyclooxygenase‐2, nitric oxide (NO), IL‐1β, IL‐6, and TNF‐α. In a rat model of acute radiation‐induced skin injury, ferulic acid significantly reduced the expression of NLRP3 inflammasome components. These inflammatory factors are also involved in rosacea pathogenesis. Additionally, ferulic acid is a potent antioxidant, terminating free radical chain reactions due to its hydroxyl and methoxy groups, acting as a free radical scavenger, enzyme inhibitor, and enhancing scavenging enzyme activity [20, 21]. Moreover, it has antimicrobial effects, inhibiting fungi in the genus *Malassezia* and protecting keratinocytes from oxidative stress and inflammatory damage, which is not possible with tetracycline antibiotics [22, 23, 24, 25]. Ferulic acid also absorbs UVB radiation (290–330 nm), enhancing sunscreen efficacy by increasing SPF and PFA [26, 27].

This study found that topical ferulic acid had an 80.00% total effective rate in treating papulopustular rosacea, significantly higher than the control group. Patients showed reduced erythema and fewer papules and pustules after 2 weeks of treatment, with optimal effects observed after 4–6 weeks. This suggests that topical ferulic acid should be administered for 2–4 weeks, with adjustments if the therapeutic effect is insufficient after 6 weeks. Ferulic acid also improved skin barrier function, indicated by increased stratum corneum hydration (SCH) and decreased transepidermal water loss (TEWL), confirming its role in improving barrier integrity, which is crucial for treating rosacea.

Adverse reactions to topical ferulic acid were mild and transient, with no impact on patient compliance or dropout rates. However, due to ferulic acid's low solubility and stability, oil‐based matrices are necessary for better absorption [28]. New encapsulation technologies like nanoemulsions, hydrogels, and phospholipid complexes can address these shortcomings [29]. The main limitation of this study was the small sample size, necessitating larger, longer clinical trials to further validate ferulic acid's efficacy and safety for rosacea treatment. There are currently no reports of direct oral or injectable use of ferulic acid in clinical practice. Most studies and applications have focused on its topical and systemic effects when used in combination with other compounds. Further research and clinical trials are necessary to fully elucidate the therapeutic potential and safety profile of ferulic acid for direct oral or injectable administration.

In summary, topical ferulic acid is an effective adjunct treatment for papulopustular rosacea, improving skin lesions and barrier function while minimizing adverse reactions. Its clinical use and promotion are recommended.

## Author Contributions

Dong Zhang and Weiyuan Ma designed the study; Xing Wang was responsible for management of the patients and drafting the manuscript; Yonghong Xue, Jingjie Zhang, Meiling Li, Wenxiu Ge, Hongzi Zhu, Zengxiang Luo and Xiangfeng Yuan participated in management of the patients and analyzed the images. The manuscript has been read and approved by all named authors.

## Ethics Statement

The patient had given written informed consent for the publication of her clinical details and accompanying images. Institutional approval is not required for this case study.

## Conflicts of Interest

The authors declare no conflicts of interest.

## Data Availability

Data supporting this study's findings are available by correspondence author Dong Zhang upon reasonable request.
